# Explainable machine learning for preoperative relapse prediction in molecularly stratified endometrial cancer: A single-center finnish cohort study

**DOI:** 10.1016/j.csbj.2025.12.018

**Published:** 2025-12-22

**Authors:** Sergio Vela Moreno, Masuma Khatun, Annukka Pasanen, Ralf Bützow, Andres Salumets, Mikko Loukovaara, Vijayachitra Modhukur

**Affiliations:** a Department of Obstetrics and Gynecology, Institute of Clinical Medicine, University of Tartu, Tartu, Estonia; b Celvia CC, Tartu, Estonia; c Helsinki University Hospital and University of Helsinki, Department of Obstetrics and Gynecology, Helsinki, Finland; d University of Helsinki, Faculty of Medicine, Helsinki University Hospital, and Research Program in Applied Tumor Genomics, Department of Pathology, Helsinki, Finland; e Department of Clinical Science, Intervention and Technology, Karolinska Institute and Karolinska University Hospital, Stockholm, Sweden, Finland; f Helsinki University Hospital and University of Helsinki, Comprehensive Cancer Center, Helsinki, Finland

**Keywords:** Endometrial cancer, Preoperative prediction, Relapse timing, Machine learning, Explainable AI (XAI), SHAP, Molecular classification, Risk stratification

## Abstract

Relapse risk in endometrial carcinoma (EC) is driven by molecular subtype, yet current WHO/ESGO classifications rely on postoperative data, limiting their preoperative use. We developed interpretable machine learning (ML) models to predict relapse timing (none, ≤6 months, >6 months) using exclusively preoperative multimodal data. In a single-center retrospective cohort of 784 EC patients, clinicopathological, molecular, immunohistochemical, and systemic biomarkers were integrated using four feature strategies: Traditional (clinicopathology), ESGO-based (guideline risk groups),TP53 + MMRd (high-risk biology), and POLE (low-risk). Random Forest (RF), Support Vector Machine, k-Nearest Neighbors, Gradient Boosting (GBM) models were trained with leakage-safe preprocessing and evaluated by area under the curve (AUC), accuracy, recall, and F1 score, with interpretability assessed by SHapley Additive exPlanations (SHAP). The RF-Traditional model achieved the best overall performance (F1 = 0.895, AUC = 0.840), while the GBM-POLE model achieved the highest sensitivity (F1 = 0.886, AUC = 0.842). However, prediction of Late Relapse remained challenging (F1 = 0.31) due to class rarity and heterogeneity. Key predictors included ARID1A loss, elevated CA125, thrombocytosis, and p16 expression among key predictors of relapse; while shared high-risk features across models were advanced stage, deep myometrial invasion, elevated CA125, and positive cytology. While multi-center validation is essential, our findings support biologically coherent predictions for individualized preoperative risk stratification, particularly for high-risk molecular subtypes.

## Highlights

• Pre-operative XAI models predict endometrial cancer relapse timing with an AUC of up to 0.842.
• Traditional models achieved 0.797 accuracy, while POLE models maximized sensitivity at 0.886.
• SHAP analysis identified stage, LVSI, size, cytology, CA125, and PR as key predictive drivers.
• Early relapse linked to burden/aggressiveness; late to spread/size; no Relapse indicates an inverse profile.
• Transparent outputs enable risk-aligned pre-operative planning and warrant multi-center validation.

## Graphical abstract

**Figure:**
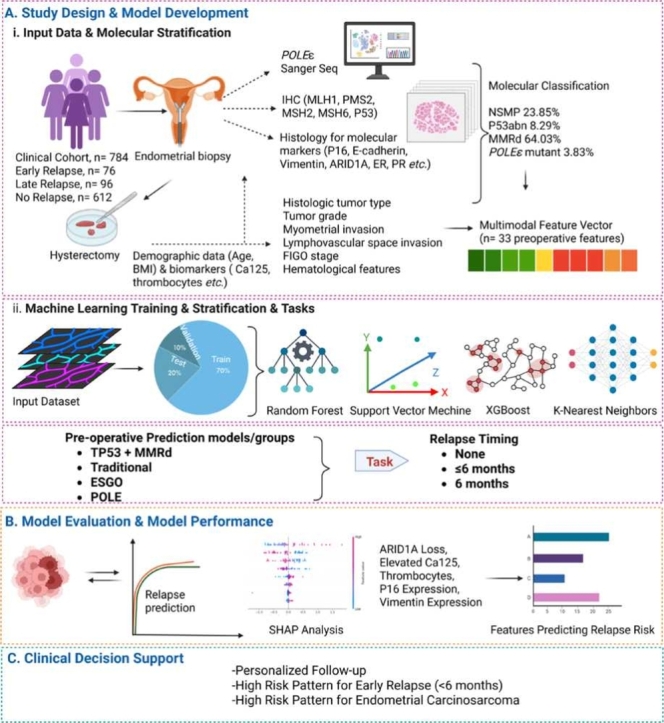

## 1 Introduction

Endometrial cancer (EC) is the most common gynaecologic malignancy in developed countries, with incidence and mortality projected to increase by 55 % by 2030 [11], [55], largely driven by obesity and metabolic syndrome [55], [67]. Despite advances in adjuvant therapies, including immunotherapy trials (RUBY, GY018, and DUO-E) [23], [78], 15–20 % of patients experience relapse, often in the vaginal vault, pelvis, peritoneum, or distant organs, with a poor prognosis and limited treatment options [16], [5], [72]. Endometrial carcinosarcoma, a rare but highly aggressive subtype accounting for 5–6 % of ECs [9], exhibits relapse rates of 40–60 % and high mortality [8].

Traditional risk stratification relies on clinicopathological features such as age, tumor grade, FIGO (International Federation of Gynecology and Obstetrics) stage, and lymphovascular space invasion (LVSI) [13], [80]. However, these parameters often lack reproducibility and prognostic accuracy, particularly in high-risk or recurrent cases [53]. Molecular classifiers, including The Cancer Genome Atlas (TCGA) and its clinical surrogate, The Proactive Molecular Risk Classifier for Endometrial Cancer (ProMisE), have refined EC stratification into four distinct subtypes: *POLE*-ultramutated (*POLE*mut, favorable, low relapse risk), mismatch repair-deficient (MMRd, higher relapse risk), p53-abnormal (p53abn, poor prognosis, higher relapse risk), and no specific molecular profile (NSMP, intermediate) [32], [36]. These are now integrated into the 2023 FIGO staging, which recognizes *POLEmut* tumors as favorable and p53abn as adverse, even in early-stage disease [24], [71].

Despite these advances, relapse prediction remains challenging, particularly in NSMP, p53abn, and carcinosarcoma subgroups [33], [75]. Existing models often fail to incorporate comprehensive molecular and clinical variables [73], while symptom-based surveillance may miss early asymptomatic relapses that are linked to poorer outcomes [2], [31]. Post-relapse survival varies significantly: 43 months for MMRd, 39 for NSMP, and only 10 for p53abn, underscoring the urgent need for improved predictive tools [34], [68]. Biomarkers (*e.g.*, ARID1A [39], p16 [48], β-catenin, E-cadherin [66], and systemic markers (e.g., CA125, platelet count) [50], [66] show promise but lack integration into multivariable risk models.

Machine learning (ML) continues to show promise in enhancing relapse prediction in EC, particularly when integrated with molecular classification. Ensemble models (e.g., AdaBoost, XGBoost, and Random Forest, RF) combined with interpretability tools like SHAP (SHapley Additive exPlanations) have boosted both predictive accuracy and clinical applicability. For instance, the TJHPEC model achieved an area under the curve (AUC) of 0.93 using routine clinical features across 1935 patients [77], while radiomics-based models leveraging preoperative CT scans reached AUCs up to 0.90 [7]. Molecularly informed models like im4MEC correlated strongly with 5-year relapse-free survival [21] and NU-CAT predicted progression and relapse with 75 % accuracy [79]. Additional approaches include Random Forest (RF)-based predictors of high-grade EC (AUC 0.85) [62], biomarker-integrated nomograms [14], deep learning on haematoxylin and eosin (H&E)-stained slides [15], [22], and the HECTOR model for distant relapse prediction [74]. However, few models have been validated in molecularly stratified cohorts, and preoperative, multi – class prediction of relapse timing, particularly in high-risk EC and carcinosarcoma, remains underexplored.

To address these gaps, we developed and compared interpretable ML models for preoperative relapse prediction of relapse timing (No Relapse, ≤6 months, >6 months) in EC, including carcinosarcoma. Four complementary approaches were implemented: 1) Traditional clinicopathological, (2) ESGO guideline-based, (3) Tp53 + MMRd biology-driven high-risk, and (4) POLE low-risk feature strategies. By integrating multimodal preoperative data, including molecular classifiers, biomarkers, and clinicopathological features, we sought to evaluate trade-offs between accuracy, sensitivity, and clinical utility, while ensuring model interpretability through SHAP (SHapley Additive exPlanations).

## 2 Materials and methods

### 2.1 Study design and patient cohort

This retrospective study included 784 patients with stage I–IV EC who underwent hysterectomy at Helsinki University Hospital between 2007 and 2013. Ethical approval was obtained from the Helsinki University Hospital Institutional Review Board (HUS/491/2021) and the Finnish Medicines Agency (FIMEA/2021/005153). Informed consent was waived for this retrospective cohort.

Clinicopathological data were retrieved from institutional records. Staging followed the FIGO 2009 guidelines [60]. Tumours were classified into molecular and clinicopathological risk groups using the ESGO/ESTRO/ESP 2021 guidelines [13]. Tumours were classified into low, intermediate, high-intermediate, high, and advanced-metastatic risk categories based on molecular subtype and clinicopathological factors, including histology, grade, depth of myometrial invasion, LVSI, and FIGO stage. The guidelines do not assign risk categories to stage I–IVA MMRd and NSMP clear cell carcinomas with myometrial invasion, or to stage III–IVA *POLE*mut tumours, due to limited supporting data. In this study, these tumors were classified as high-risk. LVSI was assessed using a three-tiered system: none, focal, or substantial [13]. Relapse status was obtained from hospital or referral centre records, and cytology from peritoneal washings taken during surgery.

### 2.2 Preoperative clinical and biomarker assessment

Preoperative data included American Society of Anaesthesiologists (ASA) physical status scores extracted from anaesthesia records and standardized to the 2014 classification system [30]. Patients who were current smokers, had a BMI of 30–40 kg/m², or had well-controlled diabetes were assigned ASA II, while those with severe obesity (BMI ≥40 kg/m²) were classified as ASA III. Hematologic parameters were obtained from pre-treatment blood count using photometry, impedance, and flow cytometry. Anaemia was defined as haemoglobin (Hb) < 117 g/L, leukocytosis as WBC > 8.2 × 10⁹/L, and thrombocytosis as platelets > 360 × 10⁹/L [51]. Serum CA125 levels were measured via chemiluminescent microparticle immunoassay on the Abbott Architect 2000i system, with values > 35 U/ML considered elevated [4]. A tumor size threshold of 25 mm was applied based on prior evidence linking it to relapse risk [69].

### 2.3 Molecular classification and immunohistochemistry

While molecular classification is traditionally performed on hysterectomy specimens, multiple studies have demonstrated that preoperative assessment is feasible using endometrial biopsy, pipelle, or curettage samples. Key molecular markers, including MMR status, p53 expression, and POLE mutations, can be reliably determined from these small preoperative specimens, enabling their integration into risk prediction models before definitive surgery [1], [52], [65]. These preoperative molecular data were incorporated into our ML models to enable individualized relapse-risk prediction before definitive surgery.

In brief, multicore tissue microarrays (four tumor cores/case) were constructed following established protocols and scanning using the 3D Histech Pannoramic 250 Flash II. Digital images were reviewed via WebMicroscope. Immunohistochemical (IHC) scoring was performed by a pathologist blinded to clinical outcomes (A.P.), with equivocal cases confirmed by a second pathologist (R.B.) [28], [29], [59]. Molecular classification followed WHO guidance, assigning tumors to MMRd, P53abn*, POLE*mut, or NSMP. In cases with overlapping features, classification was based on the prognostically dominant alteration [25], [35], [57], [58]. Fresh-frozen tumor samples were collected for POLE mutation analysis, with exonuclease-domain hotspot mutations confirmed by targeted sequencing (exon 9, 13, 14) [56]. IHC panels, scoring thresholds, and assay details are summarized in Supplementary Table S1.2.4

### 2.4 **Explainable machine learning framework**

To predict relapse risk, supervised machine learning pipelines were implemented to assess relapse risk, categorized into multi-class, namely, No Relapse, ≤ 6 months, or > 6 months. Data was pre-processed by eliminating features with > 30 % missing values, and only pre-operative variables were retained. Missing values were imputed using median/mode substitution with the *na.roughfix* function in the R package *randomForest*. Variables were one-hot encoded, normalized, and outliers capped using interquartile range (IQR) thresholds. After this preprocessing, the final feature sets were selected for every molecular dataset (28–29 variables depending on the molecular dataset).

To capture different perspectives of relapse prediction, four complementary models were developed. The Traditional model incorporated established clinicopathological features (FIGO stage, grade, histology, LVSI, tumor size, receptor status) and served as the benchmark reflecting current practice. The ESGO-based model applied the 2021 ESGO/ESTRO/ESP risk classification as a guideline-based comparator. The TP53 + MMRd model targets two molecularly defined high-risk subgroups (TP53-abnormal and MMR-deficient) with a compact, biology-driven feature set. Finally, the POLE model focused on the biologically distinct *POLE*mut subgroup, typically associated with excellent prognosis, to assess whether subgroup-specific modeling improved discrimination.

For each strategy (Traditional, ESGO, TP53 + MMRd, POLE), a dedicated data frame was generated and split into 70:20:10 train-test-validation sets. Recursive feature elimination (RFE) was performed using 5-fold cross-validation (CV), optimizing for the metric F1 score in risk prediction, given the significant class imbalance between classes. Feature selection was implemented manually using the *tidymodels* R package. Tumor size thresholds were iteratively optimized to improve accuracy [69].

Additionally, due to the lack of an external validation cohort, temporal validation was implemented. To do this, the cohort was divided into older samples (collected from 2007 to 2011) and newer ones (collected in 2012). The older sample set was split into an 80:20 train-test partition, while the newer samples were used as the validation set. The feature selection procedure mirrored that used for the main model.

### 2.5 Model development and evaluation

Four supervised classification algorithms were evaluated: Random Forest (RF), Support Vector Machines (SVM), k-Nearest Neighbors (KNN), and Gradient Boosting (GBM). Models were optimized using grid search with 10-fold stratified cross-validation. Class imbalance was addressed through SMOTE, under-sampling, and oversampling, which were implemented inside the cross-validation folds using the *sampling* flag in the *trainControl* function of the package *caret*.

All models for relapse risk were optimized for AUC, a threshold-invariant metric that minimizes the effect of class imbalance. Evaluation metrics included:

Accuracy = (TP + TN) / (TP + TN + FP + FN)

Recall/Sensitivity = TP / (TP + FN)

F1-score = 2TP / (2TP + FP + FN)

• AUC for each class, calculated as the integral under the ROC curve (representation of True Positive Rate (TPR) (TP / (TP + FN)) and False Positive Rate (FPR) (FP / (FP + TN))).
• Precision-Recall area under the receiver operating characteristic curve (PR-AUC) for each class, calculated as the integral under the Precision-Recall curve (representation of Precision (TP / (TP + FP)) and Recall (TP / (TP + FN)).

TP=True Positive, FP=False Positive, TN=True Negative, FN=False Negative

All models were implemented in R 4.5.0 using caret 7.0–1, randomForest 4.7–1.2, dplyr 1.1.2, gbm 2.2.2, kernelshap 0.7.0, and shapviz 0.9.7. Analyses were performed on a 64-core Intel Xeon server (256 GB RAM, Ubuntu 20.04 LTS).

### 2.6 XAI-based model interpretability

To enhance clinical trust and applicability, SHAP was employed to quantify feature contributions to predictions [45]. SHAP values were computed using kernelshap with k-means background sampling (m=50, 1000 samples per observation). Class-specific SHAP values were visualized using shapviz, enabling identification of key predictors for each relapse category (Early, Late, No Relapse). Implementations were performed using R packages *kernelshap* and *shapviz*. Feature importance stability through SHAP was evaluated using a personalized function and the package *Hmisc*. A summary of the ML algorithms and parameters is shown in Table 1**.** An overview of the ML pipeline is shown in Supplementary Figure S1**.**

**Table 1:** Summary of the machine learning algorithm.

**Component**	**Settings**
Data partitions	Stratified split per strategy: 70 % train, 20 % test, 10 % hold-out validation; seed 123.
Missingness	Drop features with > 30 % missingness. Median (numeric), mode (categorical).
Preprocessing	Impute median/mode (na.roughfix) → one-hot encode → z-score for SVM/KNN (trees unscaled) → winsorize at 1st/99th pct (sens.: 1.5 ×IQR) → remove near-zero-variance/collinear dummies → evaluate tumor-size cut-points 20/25/30/35 mm; retain 25 mm.
Feature selection	RFE with 10-fold stratified CV optimizing macro-F1; RFE inside CV.
Imbalance handling (in-fold)	SMOTE (k = 5, target ≈1:1), random undersampling (retain 60–80 % majority), random oversampling (1.5–3 × minority); pick per algorithm by CV macro-F1.
Algorithms & grids	RF: ntree {500,1000}, mtry {1:Feature Number}, nodesize {1,5}. SVM-RBF: C {2^(-1:2)}, γ {2^(-2:1)}. KNN: k {3,5,7,9,11,13,15,17,19,21}. GBM: trees {50:1500}, depth {1,5,9}, shrinkage {0.01,0.05,0.1}, min_obs_node {5,10,20}, subsample {0.5,0.8,1.0}.
Tuning	Grid search with 10-fold stratified CV; common seed across folds.
SHAP (interpretability)	kernelshap background m= 50 (k-means on training), nsamples= 1000/obs, link=logit; class-specific SHAP (No/Early/Late); global importance = mean |SHAP| stacked by class; plots via shapviz 0.9.7.
Software & hardware	R 4.5.0; key packages: readr (v2.1.5), dplyr (v1.1.4), caret (v7.0.1), randomForest (v4.7.1.2), tidyverse (v2.0.0), finalfit (v1.0.8), tidymodels (v1.3.0), vip (v0.4.1), themis (v1.0.3), MLmetrics (v1.1.3), yardstick (v1.3.2), ggplot2 (v3.5.2), tibble (v3.2.1), purrr (v1.0.1), rlang (v1.1.6), gbm (v2.2.2), pROC (v1.18.5), PRROC (v1.4), kernelshap (v0.9.0), shapviz (v0.9.7), reshape2 (v1.4.4), ggVennDiagram (v1.5.3); 64-core Intel Xeon, 256 GB RAM, Ubuntu 20.04 LTS.

## 3 Results

### 3.1 Patient cohort and molecular stratification

A total of 784 patients with EC were included, of whom 172 (22 %) experienced relapse — 76 Early (≤6 months) and 96 Late (>6 months) relapse. The remaining 612 patients (78 %) remained relapse-free.

Patients were stratified into four molecular subgroups: MMRd (64.0 %), NSMP (23.9 %), p53abn (8.3 %), and *POLEmut* (3.8 %). Thirty-three preoperative features were integrated into a multimodal feature vector for ML analyses **(Graphical abstract**).

Relapsed cases were significantly enriched in advanced FIGO stages (II–IV), non-endometrioid histology (serous and carcinosarcoma), and positive LVSI. Tumors > 25 mm, positive peritoneal cytology, deep myometrial invasion, and molecular alterations (p53abn, MMRd) were more frequent in relapsed patients. On the other hand, *POLEmut* tumors showed the lowest relapse rates. Additional relapse-associated features included p16 positivity, E-cadherin loss, vimentin expression, ARID1A loss, elevated CA125, and increased thrombocyte and leucocyte counts. Full demographic and clinicopathological comparisons are presented in Table 2.

**Table 2:** Demographic and clinicopathological characteristics of patients (n = 784).

**Label**	**Levels**	**Early Relapse (n = 76, 10.7 %)**	**Late Relapse (n = 96, 12.2 %)**	**No Relapse (n = 612, 78.1 %)**	**P**
2021 Molecular ESGO/ESTRO/ESP*	Molecular low risk	4 (5.3)	20 (20.1)	299 (48.9)	**< 0.001**
	Molecular intermediate risk	1 (1.3)	9 (9.4)	56 (9.2)	
	Molecular high-intermediate risk	6 (7.9)	18 (18.8)	56 (9.2)	
	Molecular high risk	27 (35.5)	28 (29.2)	76 (12.4)	
	Advanced-metastatic	19 (25.0)	4 (4.2)	0 (0.0)	
Applied Molecular Classification*	NSMP	14 (18.4)	21 (21.9)	152 (24.8)	**< 0.001**
	MMRd	24 (31.6)	46 (47.9)	271 (44.3)	
	*POLE*mut	1 (1.3)	0 (0.0)	29 (4.7)	
	p53abn	18 (23.7)	12 (12.5)	35 (5.7)	
p53abn	Aberrant (negative or strong)	10 (13.2)	19 (19.8)	105 (17.2)	0.515
	Wild type	66 (86.8)	77 (80.2)	507 (82.8)	
MMRd	Deficient	30 (39.5)	35 (36.5)	217 (35.5)	0.785
	Proficient	46 (60.5)	61 (63.5)	395 (64.5)	
POLE*	Normal	55 (72.4)	57 (59.4)	366 (59.8)	0.798
	Mutated	4 (5.3)	5 (5.2)	23 (3.8)	
Stage I vs II-IV	Stage I	17 (22.4)	56 (58.3)	526 (85.9)	**< 0.001**
	Stage II-IV	59 (77.6)	40 (41.7)	86 (14.1)	
Histology and Grade	Endometrioid G1–2	24 (31.6)	64 (66.7)	509 (83.2)	**< 0.001**
	Endometrioid G3	22 (28.9)	13 (13.5)	66 (10.8)	
	Non-endometrioid	30 (39.5)	19 (19.8)	37 (6.0)	
LVSI	No	35 (46.1)	50 (52.1)	511 (83.5)	**< 0.001**
	Yes	41 (53.9)	46 (47.9)	101 (16.5)	
Myometrial Invasion	Mean (SD)	63.2 (31.1)	51.3 (31.5)	35.3 (27.4)	**< 0.001**
Cytology	Negative	51 (67.1)	82 (85.4)	596 (97.4)	**< 0.001**
	Positive	25 (32.9)	13 (13.5)	12 (2.0)	
	Positive for ovarian carcinoma	0 (0.0)	1 (1.0)	4 (0.7)	
Tumor Size*	< 25 mm	8 (10.5)	21 (21.9)	262 (42.8)	**< 0.001**
	> 25 mm	65 (85.5)	74 (77.1)	341 (55.7)	
Diabetes Mellitus	No	63 (82.9)	78 (81.2)	497 (81.2)	0.938
	Type 2	13 (17.1)	18 (18.8)	114 (18.6)	
	Type 1	0 (0.0)	0 (0.0)	1 (0.2)	
BMI	Mean (SD)	27.7 (6.2)	28.4 (6.8)	28.6 (6.1)	0.473
Age	Mean (SD)	70.9 (11.9)	68.4 (10.7)	67.0 (10.3)	**0.007**
Smoker	No	68 (89.5)	74 (77.1)	492 (80.4)	0.189
	Yes	4 (5.3)	10 (10.4)	70 (11.4)	
	Former	4 (5.3)	12 (12.5)	50 (8.2)	
ASA Score	1	1 (1.3)	8 (8.3)	27 (4.4)	**0.018**
	2	21 (27.6)	35 (36.5)	256 (41.8)	
	3	43 (56.6)	43 (44.8)	286 (46.7)	
	4	11 (14.5)	10 (10.4)	43 (7.0)	
Thrombocyte	Mean (SD)	283.4 (93.2)	276.4 (84.5)	258.3 (72.5)	**0.005**
Leucocyte	Mean (SD)	8.0 (2.7)	7.4 (2.2)	6.9 (2.1)	**< 0.001**
Hemoglobin	Mean (SD)	125.5 (17.8)	131.2 (15.7)	133.7 (14.8)	**< 0.001**
CA125	Mean (SD)	235.2 (540.9)	120.3 (339.0)	43.1 (128.9)	**< 0.001**
PD-L1 expression*	< 1 %	65 (85.5)	84 (87.5)	509 (83.2)	0.013
	1–4 %	4 (5.3)	6 (6.2)	27 (4.4)	
	5–9 %	3 (3.9)	0 (0.0)	3 (0.5)	
	10–49 %	2 (2.6)	1 (1.0)	12 (2.0)	
	50 % or more	0 (0.0)	2 (2.1)	1 (0.2)	
ARID1A	Positive	61 (80.3)	76 (79.2)	473 (77.3)	0.793
	Negative	15 (19.7)	20 (20.8)	139 (22.7)	
Estrogen Receptor (ER)*	ER under 1 %	32 (42.1)	17 (17.7)	49 (8.0)	**< 0.001**
	ER 1 % or more	42 (55.3)	78 (81.2)	516 (84.3)	
Progesterone Receptor (PR)*	Negative	31 (40.8)	34 (35.4)	81 (13.2)	**< 0.001**
	Positive (>10 %)	39 (51.3)	61 (63.5)	482 (78.8)	
HER2*	Negative	70 (92.1)	93 (96.9)	559 (91.3)	**0.001**
	Positive	4 (5.3)	2 (2.1)	3 (0.5)	
Beta-catenin*	Negative	65 (85.5)	85 (88.5)	511 (83.5)	0.824
	Positive	9 (11.8)	10 (10.4)	56 (9.2)	
HNFβ	Negative	60 (78.9)	85 (88.5)	558 (91.2)	**0.001**
	Positive	16 (21.1)	11 (11.5)	54 (8.8)	
p16	Positive	73 (96.1)	96 (100.0)	600 (98.0)	0.169
	Negative	3 (3.9)	0 (0.0)	12 (2.0)	
E-cadherin	Normal	32 (42.1)	42 (43.8)	408 (66.7)	**< 0.001**
	Loss	13 (17.1)	7 (7.3)	15 (2.5)	
	Weakened	31 (40.8)	47 (49.0)	189 (30.9)	
Vimentin	Positive	50 (65.8)	75 (78.1)	522 (85.3)	**< 0.001**
	Negative	26 (34.2)	21 (21.9)	90 (14.7)	
CD171*	Negative	47 (61.8)	73 (76.0)	501 (81.9)	**< 0.001**
	Positive	27 (35.5)	22 (22.9)	60 (9.8)	

* Missing data presented as per “Total number per: Early, Late, No Relapse”: 2021 Molecular ESGO/ESTRO/ESP (161: 19 [25.0 %], 17 [17.7 %], 125 [20.4 %]); Applied Molecular classification (161: 19 [25.0 %], 17 [17.7 %], 125 [20.4 %]); *POLE* status (374: 17 [22.4 %], 34 [35.4 %], 223 [36.4 %]); Tumor size (13: 3 [3.9 %], 1 [1.0 %], 9 [1.5 %]); PD-L1 expression (65: 2 [2.6 %], 3 [3.1 %], 60 [9.8 %]); ER (54: 2 [2.6 %], 1 [1.0 %], 47 [7.7 %]); PR (66: 6 [7.9 %], 1 [1.0 %], 49 [8.0 %]); HER2 (57: 2 [2.6 %], 1 [1.0 %], 50 [8.2 %]); Beta-catenin (54: 2 [2.6 %], 1 [1.0 %], 45 [7.4 %]); CD171 (54: 2 [2.6 %], 1 [1.0 %], 51 [8.3 %]). P-values were unadjusted due to the descriptive representation of baseline variables.

Graphical abstract. Workflow for Machine Learning (ML)–based relapse prediction in endometrial cancer. The schematic figure outlines the study pipeline from patient inclusion to clinical application. (A) A retrospective cohort of 784 EC patients was analyzed, integrating clinical, demographic, biomarker, and molecular data into a multimodal feature set. Patients were stratified into four molecular subgroups: NSMP, p53abn, MMRd, and *POLE*mut. Multiple ML algorithms (Random Forest, SVM, XGBoost, k-NN) were trained to predict relapse timing. (B) Model performance was evaluated using area under the curve (AUC) and accuracy metrics, with SHapley Additive exPlanations (SHAP) analysis applied to identify key predictive features across models. (C) SHAP-based interpretation was used to support individualized relapse risk stratification, enabling potential clinical decision-making for surveillance and therapy.

### 3.2 Feature selection and model inputs

Recursive Feature Elimination (RFE) identified the key preoperative features for each model, with systemic biomarkers (CA125, thrombocytes, leucocytes) and invasion-related variables (myometrial depth, LVSI, cytology) consistently ranked highest. Traditional risk modifiers (BMI, diabetes, smoking) were rarely selected. For the risk of relapse, the TP53 + MMRd model achieved a theoretical F1 score of 0.512 using 19 features, including myometrial invasion, CA125, advanced stage (II–IV), thrombocyte count, and histology. Similar performance was observed with the applied molecular classification (F1 score = 0.492, 22 features) and the ESGO molecular classification (F1 score = 0.528, 23 features), where overlapping predictors included CA125, myometrial invasion, stage, and leucocyte count. The *POLE*-specific model achieved the highest F1 score among single-subtype analyses (0.557 with 28 features), although feature selection was limited by the sample size of *POLE*mut tumours. In summary, across all analyses, systemic biomarkers (CA125, thrombocytes, leucocytes) and invasion-related variables (myometrial depth, LVSI, cytology) consistently emerged among the top-ranked features, underscoring their value for preoperative risk stratification. In contrast, traditional risk modifiers such as BMI, diabetes, and smoking status were rarely selected, suggesting their limited predictive contribution in molecularly stratified cohorts. Following feature selection, the optimal subsets of covariates retained to explain relapse risk were as follows: 19 for the TP53 + MMRd, 22 for the Traditional, 28 for the POLE, and 23 for the ESGO-based model (Supplementary Table S2). The results of RFE stability are provided in Supplementary Table S3. For improved clarity, these results are also visualized as a Heatmap in Supplementary Figure S2.

### 3.3 Model metrics across the predictive model

**Fig. 1:**
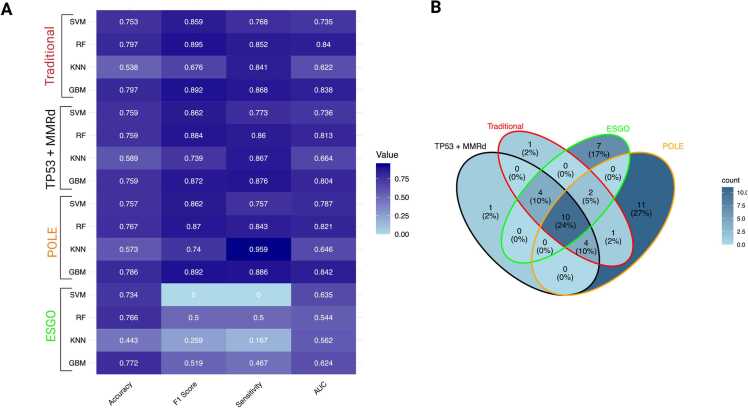
Performance Metrics and Feature Overlap Across Predictive Models. (A) Heatmap showing performance metrics (Accuracy, F1 Score, Sensitivity, AUC) for four machine learning algorithms (SVM, RF, KNN, GBM) across molecular subgroups: Traditional (red), TP53 + MMRd (black), POLE (orange), and ESGO (green). Darker shades indicate higher metric values. (**B)** Venn diagram illustrates the distribution of overlapping features among the four molecular subgroups. Each segment is annotated with case counts and their percentage representation. A blue gradient indicates density, highlighting both unique and shared cases across subgroup intersections.

The performance of four ML algorithms (SVM, RF, KNN, GBM) across the four stratification strategies. Performance variability by molecular subgroup was evident, with stronger discrimination in POLE models and more modest performance in ESGO-based stratification **(**Fig. 1**A).** Feature overlaps across models, illustrated in Fig. 1**B**, reveal both shared and subgroup-specific predictors.

### 3.4 Model performance comparison

Performance metrics for the above-mentioned ML models are summarized in Table 3 and visualized in Fig. 1**A**. The POLE model demonstrated the highest discrimination (macro-averaged AUC 0.842) and sensitivity (0.886). In contrast, the Traditional model achieved the best overall accuracy (0.797) and tied with POLE for the top F1 score (0.892), while requiring fewer predictors (22 vs. 28). The TP53 + MMRd model utilized the fewest predictors (19) and delivered competitive sensitivity (0.876) and F1 score (0.872), albeit with slightly lower accuracy (0.759) and AUC (0.804). By contrast, the ESGO- guideline-based model underperformed, with lower discrimination (AUC 0.624) and F1 score (0.519), despite a comparable feature count (23). In summary, these findings highlight a trade-off: the Traditional model delivers a parsimonious and well-balanced solution, whereas the POLE model lays emphasis on sensitivity and discrimination. The TP53 + MMRd model offers a compact, intermediate option. However, when assessing robustness against temporal dataset shift, the Traditional model, trained on the early cohort (2007–2011) and tested on the later, held-out cohort (2012), demonstrated a notable decrease in generalizability. This temporal validation resulted in a Macro-averaged AUC of 0.69 (95 % CI 0.46–0.93) and a Macro-F1 score of 0.48 (95 % CI 0.29–0.70). The decline in performance is linked to temporal dataset shifts due to advancements in molecular testing and changes in clinical management practices throughout the study duration, highlighting the constraints of a retrospective, single-center design for long-term external generalization. Future updates will incorporate PR-AUC and balanced accuracy once harmonized per-class outputs are accessible. The detailed data for all predictive models with all four ML algorithms are outlined in Supplementary Table S4**.**

**Table 3:** Performance of pre-operative predictive models, including confidence interval (CI) optimized with the best Gradient Boosting (GBM) algorithm.

**Model**	**Accuracy**	**F1 Score**	**Sensitivity**	**AUC**	**Features, n**
TP53 + MMRd	0.759 (Cl 0.64–0.82)	0.872 (Cl 0.77–0.91)	0.876 (Cl 0.73–0.92)	0.804 (Cl 0.71–0.98)	19
Traditional	0.797 (CI 0.62–0.80)	0.892 (CI 0.78–0.92)	0.868 (CI 0.70–0.90)	0.838 (CI 0.70–0.99)	22
ESGO	0.772 (CI 0.64–0.81)	0.519 (CI 0.21–0.73)	0.467 (CI 0.17–1.00)	0.624 (CI 0.51–0.96)	23
POLE	0.786 (CI 0.75–0.84)	0.892 (CI 0.80–0.93)	0.886 (CI 0.77–0.97)	0.842 (CI 0.76–0.99)	28

### 3.5 Overlap and feature distribution across models

Venn diagram analysis revealed overlapping and unique case distributions across models (Fig. 1**B**). The POLE group had the highest number of cases (11), characterized by features such as LVSI, large tumor size, higher ASA score and BMI, variable PD-L1 expression (1–>10 %), diabetes mellitus, and positivity for β-catenin, HNFβ, and vimentin. The ESGO group included 7 cases, mainly showing advanced or metastatic status, thrombocytosis, molecular high or intermediate risk, ER positivity, loss of E-cadherin, and a history of smoking. The TP53 + MMRd and Traditional groups each had 1 case, associated with proficient MMRd and p53abn, respectively.

Among overlapping categories, Traditional/POLE (1 case) showed positive ARID1A expression. The TP53 + MMRd/Traditional/ESGO group (4 cases) demonstrated LVSI, higher BMI and ASA scores, and larger tumor size. Similarly, TP53 + MMRd/Traditional/POLE (4 cases) was characterized by ER positivity, thrombocytosis, normal E-cadherin, and non-smoking status. The Traditional/ESGO/POLE overlap (2 cases) involved endometrioid G3 tumors with weakened E-cadherin. The most complex intersection, TP53 + MMRd/Traditional/ESGO/POLE (10 cases), showed high-risk clinicopathologic features including stage II–IV disease, myometrial invasion, elevated CA125, positive CD171 and cytology, older age, leukocytosis, PR positivity, low hemoglobin, and non-endometrioid histology. Full details about the feature are presented in Supplementary Table S5**.**

### 3.6 Traditional model: class-specific performance

**Fig. 2:**
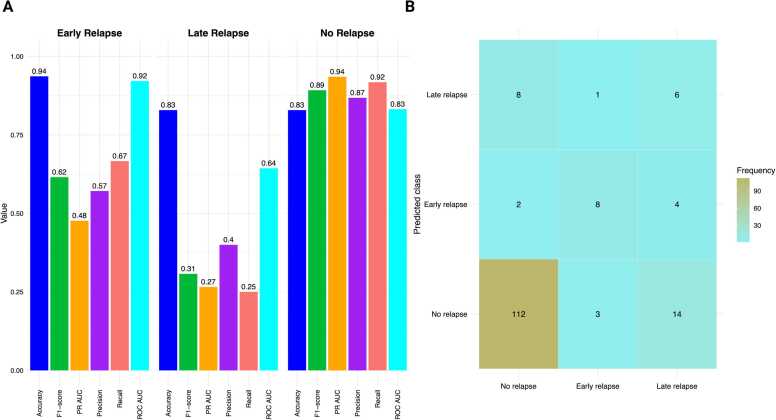
Class-Level Performance and Confusion Matrix for the Traditional Model. (A) Grouped bars display accuracy, F1, PR-AUC, precision, recall, and ROC-AUC for Early Relapse, Late Relapse, and No Relapse classes. The highest performance is observed for the No-Relapse class (F1 0.892; PR-AUC 0.935), intermediate for the Early Relapse class (F1 0.615; PR-AUC 0.477), and lowest for the Late Relapse class (F1 0.308; PR-AUC 0.266). These patterns reflect both the underlying class imbalance and the greater challenge of distinguishing Late Relapse events. (B) Confusion matrix for relapse stages (No-Relapse, Early Relapse, Late Relapse) with row-normalized recall percentages. Rows indicate true labels, while columns indicate predicted labels. The recall is strong for No-Relapse, moderate for Early Relapse (often misclassified as No-Relapse), and weak for Late Relapse (mostly misclassified as No-Relapse). Darker colors indicate a higher frequency of recall.

Per-class performance metrics for the traditional models revealed the expected imbalance pattern (Fig. 2**A)**. The model demonstrated optimal performance for the No-Relapse class (F1 =0.892, precision=0.868, recall=0.918, PR-AUC=0.935; accuracy=0.829, ROC-AUC=0.849). Performance for the Early Relapse class was moderate (F1 =0.615, precision=0.571, recall=0.667, PR-AUC=0.477; accuracy=0.937, ROC-AUC=0.913), reflecting reasonable detection with some false positives. The Late-Relapse class posed the greatest challenge (F1 =0.308, precision=0.400, recall=0.250, PR-AUC=0.266; accuracy=0.829, ROC-AUC=0.641), consistent with class rarity and overlap with other phenotypes. Overall, these results support the importance of reporting PR-AUC and F1 alongside ROC-AUC, and they suggest the implementation of targeted strategies, such as rebalancing or multimodal features, to enhance minority-class detection. Per-class performance of the Traditional model across relapse timing is presented in Supplementary Table S6**.**

The confusion matrix corroborates these class-specific metrics (Fig. 2**B**). Notably, the 'No-Relapse' category predominantly occupies the diagonal, with most instances correctly classified. The 'Early Relapse' category exhibits a moderate true-positive block, with most remaining errors misclassified as 'No-Relapse.' The 'Late Relapse' category has the least populated true-positive cell, with misclassifications primarily occurring as 'No-Relapse' and, to a lesser extent, as 'Early Relapse.' This asymmetric error pattern (Late → Early/No) aligns with class imbalance and the lower recall for late events, whereas early events are detected with greater reliability, and 'No-Relapse' is identified with high recall.

### 3.7 XAI-based model interpretability: traditional model

**Fig. 3:**
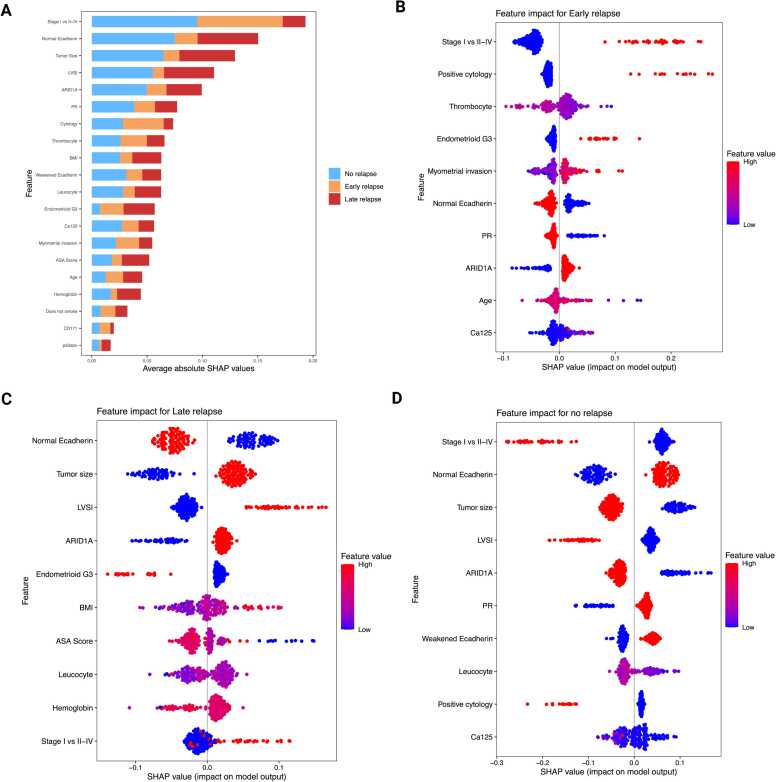
SHAP-Based Interpretation of Relapse Risk in the Traditional Model. (A) Global SHAP summary plot showing mean absolute SHAP values, ranked by feature importance and stacked by relapse class. This visualization highlights the most influential predictors across all classes. (B–D) Class-specific SHAP beeswarm plots for Early Relapse, Late Relapse, and No Relapse, respectively. Each point represents an individual patient; color indicates the feature value (red = high, blue = low), while horizontal position reflects the magnitude and direction of the feature’s impact on the predicted class probability. These plots illustrate how specific features contribute to relapse risk stratification at the patient level.

SHAP analyses demonstrated class-specific patterns that aligned with the overall feature ranking. The global importance was highlighted by the mean absolute SHAP values (organized by class), which pinpointed FIGO stage, E-cadherin status, tumor size, and LVSI as the primary predictors. These were followed by ARID1A, PR, and peritoneal cytology. There were also smaller contributions from hematologic/host factors such as thrombocytes, BMI, leucocytes, and hemoglobin, as well as CA125, myometrial invasion, and ASA score. Notably, these variables had varying levels of influence across different classes, as illustrated by the stacked bar profiles **(**Fig. 3**A).**

Early Relapse (≤6 months) was primarily driven by higher stage (II–IV), positive peritoneal cytology, thrombocytosis, grade-3 endometrioid histology (G3), deep myometrial invasion, and ARID1A loss, with elevated CA125 providing additional risk. Protective features included PR positivity (>10 %), preserved E-cadherin, and smaller tumor size **(**Fig. 3**B).** Late-relapse class (>6 months) was most strongly linked to LVSI positivity, larger tumor size, and higher stage, supported by ARID1A loss, G3 endometrioid histology, and host factors such as higher BMI/ASA, leukocytosis, and lower hemoglobin. In the Late-Relapse class, Stage I status, preserved E-cadherin, and smaller tumor size served as protective features, each contributing negative SHAP values. The latter implies that they decreased the model’s log-odds and predicted probability of Late Relapse (shifting probability toward Early/No Relapse) when other inputs were held constant **(**Fig. 3**C)**. In the No-Relapse category, factors that favored predictions of No Relapse included Stage I disease, preserved E-cadherin, PR levels above 10 %, low CA125, negative cytology, absence of LVSI, and smaller tumors. In contrast, Stage II–IV, positive LVSI, ARID1A loss, larger tumors, and positive cytology decreased the likelihood of remaining free from recurrence. Early Relapse is associated with tumor burden and biological aggressiveness, such as advanced stage, positive cytology, elevated CA125, and invasion/size. Late Relapse is more closely linked to anatomic spread and size, including advanced LVSI, larger diameter, and higher stage, while No-Relapse reflects the opposite profile **(**Fig. 3**D)**. Collectively, the panels provide a coherent and clinically intuitive distinction of relapse phenotypes. Early Relapse is primarily driven by tumour biology and burden, while Late Relapse is more associated with anatomic spread and size. In contrast, the No-Relapse profile reflects the inverse of these features. This alignment between SHAP-based interpretability and established clinical expectations offers clinical plausibility and bolsters confidence in the model's predictive framework.

### 3.8 POLE model: class-specific performance

**Fig. 4:**
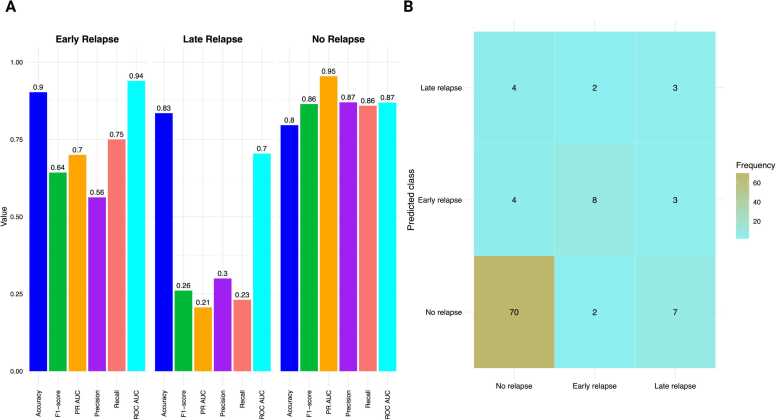
Class-Level Performance and Confusion Matrix for the POLE Model. (A) Grouped bar chart displaying performance metrics — Accuracy, F1 Score, Precision-Recall AUC (PR-AUC), Precision, and Recall for each relapse class: Early Relapse, Late Relapse, and No Relapse. The model showed highest performance for the No-Relapse class (F1 = 0.861; PR-AUC = 0.945), moderate performance for Early Relapse (F1 = 0.642; PR-AUC = 0.696), and lowest for Late Relapse (F1 = 0.262; PR-AUC = 0.208). These results reflect both class imbalance and the increased difficulty in identifying Late Relapse events. (B) Confusion matrix for the POLE model predictions (Early Relapse, Late Relapse, No Relapse), displaying row-normalized recall percentages. Rows correspond to true labels and columns to predicted labels. Recall is strongest for No Relapse, moderate for Early Relapse (often misclassified as No Relapse), and lowest for Late Relapse, which are commonly misclassified as Early or No Relapse. Darker shading indicates higher recall frequency.

Per-class performance metrics for the POLE model revealed a performance pattern consistent with the traditional model, largely influenced by class imbalance **(**Fig. 4**A).** The model achieved its strongest performance for the No-Relapse class (F1 = 0.892, Precision = 0.886, recall = 0.897, PR-AUC = 0.944; accuracy = 0.835, ROC-AUC = 0.869), indicating stable generalization and consistent identification of patients without relapse. The Early Relapse class showed moderate discrimination (F1 = 0.593, Precision = 0.533, recall = 0.667, PR-AUC = 0.649; accuracy = 0.893, ROC-AUC=0.939), indicating balanced sensitivity and precision, with a modest improvement in PR-AUC, compared to the traditional model. The Late Relapse class exhibited the lower performance (F1 = 0.273, Precision = 0.333, recall = 0.231, PR-AUC = 0.208; accuracy = 0.845, ROC-AUC= 0.704), reflecting continued limitations in minority-class detection. Collectively, these findings reinforce the need for targeted modeling strategies such as temporal reweighting or synthetic data augmentation to enhance recognition of rare relapse patterns, particularly late events. Per-class performance of the POLE model across relapse timing is presented in Supplemental Table S6**.**

The confusion matrix for the POLE model (Fig. 4**B**) illustrates the class-specific trends. Notably, the recall was strongest for the No-Relapse class, moderate for Early Relapse (often misclassified as No Relapse), and weakest for Late Relapse (frequently confused with both Early and No Relapse). The dominance of correct No-Relapse predictions forms a dense diagonal cluster, while the asymmetric misclassification of Late Relapse underscores both data imbalance and model conservatism. Despite this, the slightly higher PR-AUC for Early Relapse suggests improved precision–recall trade-offs relative to the traditional model.

### 3.9 XAI-based model interpretability: POLE model

**Fig. 5:**
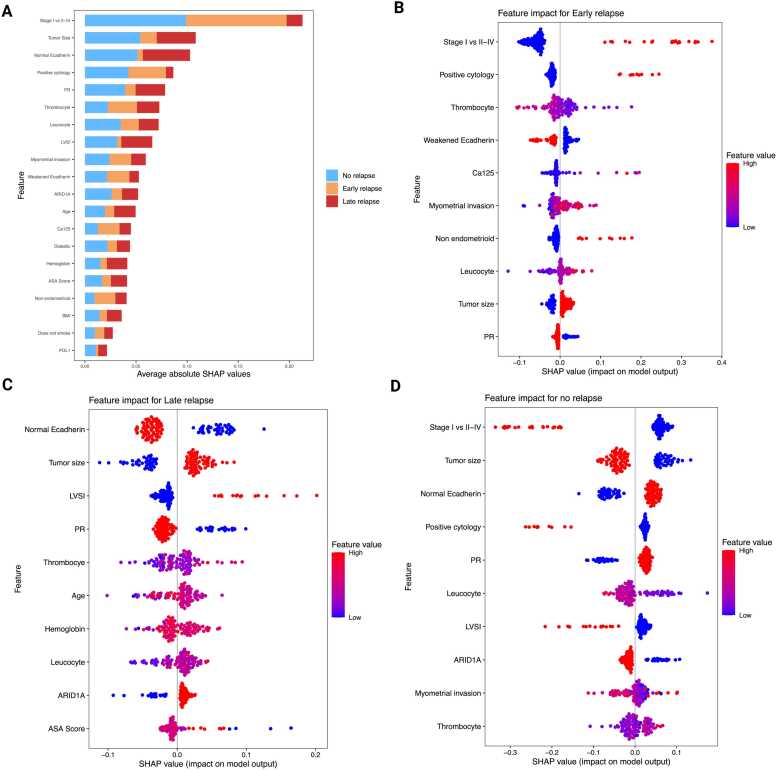
SHAP-Based Interpretation of Relapse Risk in the POLE Model. (A) Global SHAP summary plot displaying mean absolute SHAP values, ranked by overall feature importance and stacked by relapse class. This visualization highlights the most influential predictors contributing to relapse risk across the POLE subgroup. (B–D) Class-specific SHAP beeswarm plots for Early Relapse, Late Relapse, and No Relapse, respectively. Each point represents an individual patient; color indicates the feature value (red = high, blue = low), while horizontal position reflects the magnitude and direction of the feature’s impact on the predicted class probability. These plots provide insight into how specific features drive model predictions at the patient level, supporting individualized risk assessment.

The SHAP patterns for the POLE models (Fig. 5) closely resemble those of the Traditional model (Fig. 3). On a global scale, FIGO stage, tumor size, LVSI, and myometrial invasion emerged as the primary contributors by mean SHAP **(**Fig. 5**A).** Conversely, CA125 and peritoneal cytology were associated with increased risk, while progesterone receptor (PR) > 10 % shifted predictions towards No Relapse **(**Fig. 5**B).** In terms of class-specific SHAP, Early Relapse was associated with higher stage, positive cytology, and elevated CA125 levels. In contrast, Late Relapse was linked to positive LVSI and larger tumor size **(**Fig. 5**C).** Tumor stage I preserved E-cadherin, and smaller tumor sizes acted as protective factors against Late Relapse, showing negative SHAP contributions that reduced the model's probability of Late Relapse and directed predictions toward No Relapse **(**Fig. 5**D).** Compared to the Traditional model, POLE exhibited a slight reweighting, highlighting the influence of size/LVSI for Late Relapse and cytology/CA125 for Early Relapse, while maintaining the early versus late pattern.

It must be noted that the *POLEmut* subgroup in our study is small (n = 30, 3.8 %) with only one relapse, highlighting the well-known ultra-favorable prognosis of POLE-ultramutated EC cases. The RFE process excluded the binary “*POLEmut* yes/no” variable from the final feature sets of all models where it was initially considered (Traditional, POLE), as standard clinicopathological variables already classify nearly all *POLEmut* cases as “No Relapse.” The class-specific ROC-AUC and PR-AUC plots for the Traditional and POLE models are shown in Supplementary Figure S3.

## 4 Discussion

### 4.1 Study overview and clinical context

This study demonstrates that preoperative multimodal data, when analyzed through interpretable ML, can effectively predict relapse risk and timing in EC. By analyzing a large, molecularly stratified cohort of 784 patients, we developed four complementary ML models integrating clinicopathological, molecular, and systemic features. These models offer actionable insights for individualized risk stratification prior to surgery or definitive histopathological staging, consistent with prior findings [10], [40].

### 4.2 Model performance and biological interpretability

Among the molecular models, the Traditional model achieved the highest overall accuracy (0.797) with balanced performance, while the POLE-based model excelled in sensitivity (0.886) and discrimination (AUC 0.842). The TP53 + MMRd model, targeting high-risk molecular subsets, maintained competitive performance with fewer variables, underscoring the biological relevance of compact, molecularly driven modeling.

In contrast, the ESGO-based classifier exhibited limited discrimination (AUC 0.624), reflecting the limitations of static risk categories in capturing the complexity of relapse heterogeneity. The ESGO/ESTRO/ESP molecular classification is primarily prognostic, relying on categorical molecular labels and excluding continuous clinicopathologic features (e.g., tumor size, myometrial invasion depth, age) [13], [27]. As a result, the ESGO-based model cannot leverage the granular clinicopathologic variables that enhance performance in the Traditional and combined models. Temporal inconsistencies in molecular profiling across years (e.g., evolving MMRd criteria, differences in p53 scoring, and delayed introduction of POLE sequencing) likely further introduce noise and reduce its predictive accuracy [17], [44].

SHAP analyses revealed that relapse timing reflects distinct biological patterns: Early Relapse is primarily driven by tumor burden and systemic inflammation (advanced stage, positive cytology, high CA125, thrombocytosis), whereas Late Relapse is associated with invasion-related features (LVSI, tumor size, ARID1A loss). Predictions of No Relapse were characterized by PR receptor positivity, preserved E-cadherin, and early-stage disease, reinforcing the biological coherence of the model outputs [3], [64]. This mechanistic interpretability enhances clinical confidence and provides actionable decision support [46], [61].

Given the prognostic homogeneity of *POLEmut* cases, *POLE* mutation status adds minimal independent predictive value beyond classical clinicopathological features (early FIGO stage, absent/subfocal LVSI, small tumor size, preserved hormone receptors, negative peritoneal cytology, etc.). Consequently, the dedicated POLE model largely recapitulates the dominant features of the Traditional model, explaining the similar SHAP patterns and lack of meaningful performance gain. This finding may align with clinical practice, where treatment de-escalation for *POLEmut* cases is guided primarily by clinicopathological criteria [31], [44].

### 4.3 Comparison with existing literature

Prior prognostic tools in EC have mainly relied on postoperative clinicopathological variables such as FIGO stage, tumor grade, and LVSI, which inadequately capture the heterogeneity of high-risk molecular subtypes [13], [53]. Incorporation of TCGA-based Molecular classifiers into WHO/ESGO/FIGO systems has markedly improved risk stratification, yet predictive accuracy for relapse remains suboptimal, particularly in NSMP, p53abn, and carcinosarcoma cases [33].

In fact, molecular context significantly influences prognosis. The ProMisE classifiers enable the practical implementation of TCGA subgroups [70], and PORTEC-3 showed that p53abn cases, while associated with poor outcomes, benefited from chemoradiotherapy over radiotherapy alone [18]. Recent cohort studies further confirmed distinct relapse patterns across molecular subtypes, reinforcing the need for molecularly informed prediction models [38], [42]. Notably, semiquantitative LVSI remains prognostically relevant within endometrioid EC regardless of molecular classification [43], suggesting that traditional histopathological markers still hold relevance when interpreted in a molecular context, consistent with our findings. Interestingly, the overlap and feature distribution across models highlight both shared and distinct prognostic profiles. Notably, the POLE group, despite its generally favorable prognosis [39], exhibited several high-risk features such as LVSI, elevated BMI and ASA scores, and variable PD-L1 expressions, suggesting that even molecularly favorable subtypes may harbor complex clinicopathologic traits. The ESGO group, enriched for advanced disease and molecular high-risk status, aligns with prior findings linking ER positivity and E-cadherin loss to aggressive behavior [48]. Overlapping categories, particularly the TP53 + MMRd/Traditional/ESGO/POLE cluster, revealed a convergence of high-risk features, including stage II–IV disease, myometrial invasion, and elevated CA125 — traits previously associated with poor outcomes [18]. These intersections underscore the value of multimodal profiling in capturing relapse risk beyond single-model stratification [10], [40].

Recent ML models such as TJHPEC [77], NU-CATS [79], and im4MEC [21] have shown promise but often lack integration of multimodal biomarkers or preoperative applicability. While HECTOR integrated whole-slide histopathology with stage across eight EC cohorts (including PORTEC trials) to deliver strong prognostic performance with therapy-relevant stratification [74], our framework complements these efforts by offering infrastructure-light, interpretable predictions at the patient level. Similarly, a large multi-institutional Israeli XGBoost model (n ≈ 1935) demonstrated feasibility with SHAP-informed relapse prediction (AUC ≈ 0.84) [54], while MRI radiomics models integrating intertumoral and peritumoral features predict relapse with decision-curve utility [37]. Beyond EC, ML-based relapse prediction in breast cancer has shown that hybrid mechanistic and ML models improve calibration for Late Relapses [49], suggesting opportunities for similar approaches in EC. To our knowledge, by incorporating systemic and immunohistochemical markers alongside molecular classification, our approach aligns with emerging evidence supporting the value of multimodal inputs for relapse prediction [26], [50]. Notably, the identification of ARID1A and p16 as key relapse predictors aligns with prior studies linking these biomarkers to aggressive EC phenotypes [39], [48] and aligns with emerging biomarker-driven ML frameworks in cancer research [3], [46], [76]

### 4.4 Interpretability and clinical implications

SHAP-based interpretability provided transparent, class-specific insights into model predictions by highlighting key biomarkers and risk factors, such as ARID1A loss, elevated CA125, thrombocytosis, p16 expression, FIGO stage, LVSI, cytology, tumor size, and E-cadherin status. These features aligned well with established molecular and staging frameworks, reinforcing the biological plausibility of the models.

Importantly, SHAP profiles enabled differentiation between relapse phenotypes. Patients flagged as high risk for Early Relapses, often driven by tumor burden and systemic inflammation, may benefit from intensified imaging or systemic therapy. Conversely, those with Late-Relapse signals, typically associated with anatomic spread, may require extended surveillance. This level of interpretability supports personalized treatment planning and enhances clinical trust in ML-based decision support.

Embedding these models into electronic health records and generating patient-level risk summaries could facilitate their integration into multidisciplinary care. This approach is consistent with evolving ESGO–ESTRO–ESP and FIGO 2023 guidelines, which increasingly emphasize molecular stratification in EC management [12], [6], [47].

For aggressive subtypes such as carcinosarcoma, which consistently showed high predicted relapse risk and distinct biomarker profiles, these models may be particularly impactful. Their clinical utility could mirror precision strategies already in use, such as trastuzumab for HER2-positive uterine serous carcinoma [19], [20], demonstrating how biomarker-informed ML tools can personalize care and improve outcomes.

Our models achieved an AUC of up to 0.842, comparable to the externally validated ENDORISK-2 framework for predicting lymph-node metastasis in EC (AUC ≈ 0.85). Both approaches highlight the value of integrating molecular and clinicopathological data in preoperative assessment. While ENDORISK-2 informs nodal management, our models focus on relapse timing, offering complementary guidance for early treatment planning and surveillance before surgery [41].

### 4.5 Strengths and limitations

The key strengths of this study include the use of a large, well-characterized cohort, exclusive reliance on preoperative data, integration of systemic, immunohistochemical, and molecular data, and explainable AI outputs ensuring clinical interpretability.

Several limitations must also be acknowledged. First, the single-center retrospective design may limit generalizability, and external multi-center validation will be essential. Notably, External validation was not feasible because no accessible cohort offers molecular data, clinical variables, and relapse annotations comparable to our own. Differences in assay platforms, preprocessing pipelines, and follow-up protocols across institutions preclude the harmonized feature alignment required for reliable validation. Moreover, Institutional Review Board and data-sharing restrictions limit access to sensitive genomic data suitable for such analyses.

Second, class imbalance, especially the smaller number of Late Relapse events, significantly affects models’ performance. Although overall discrimination was reasonable, F1-scores for Late Relapse remained low, reflecting both the intrinsic rarity and biological heterogeneity of delayed recurrence. This minority-class signal remained limited even after applying SMOTE and undersampling, and the multinomial classification framework could not fully capture the underlying time-to-event complexity. Similarly, the small size of the *POLEmut* subgroup limits the ability to detect relapse-related signals, constraining the ML model’s potential to evaluate the independent impact of POLE status.

Third, while SHAP improves interpretability, it is sensitive to feature selection and model architecture, which may influence the stability and generalizability of feature importance rankings [63].

Finally, although the findings are promising, they do not yet support preoperative decision-making or management changes. Despite advances in molecular and genomic technologies that make preoperative data increasingly accessible, their use in routine clinical practice remains limited. Meaningful clinical application will require prospective evaluation, external validation, and integration studies to assess workflow feasibility and impact.

### 4.6 Conclusions and future directions

We developed preoperative, interpretable ML models capable of predicting relapse timing in EC, aligning with molecular risk frameworks. Stage, LVSI, tumor size, myometrial invasion, CA125, cytology, and hormone-receptor status emerged as key predictors across algorithms, showing clinically coherent effects. Gradient boosting with SHAP explanations provides patient-level transparency, supporting decision-making for surveillance and adjuvant therapy, particularly in molecularly adverse groups and aggressive histology.

Although our single-center data may limit generalizability, future research will focus on prospective, multi-center validation with standardized preoperative molecular and clinicopathological data. External validation will assess model calibration and discrimination across various populations and healthcare settings. Harmonizing preoperative sample processing and biomarker assays is crucial for wider clinical application. To enhance Late Relapse prediction, studies will explore survival-based modeling, integrating longitudinal variables, cost-sensitive learning, temporal external validation, and expanding multi-center cohorts to increase event counts and better represent late relapse cases. Moreover, integrating clinicopathological data with radiomics, liquid biopsy, and whole-slide pathology could enhance predictive accuracy (e.g., HECTOR-like and radiomics pipelines) and enable trial-based validation [37], [74]. Incorporating relapse site prediction, such as regional versus distant relapse, may further refine surveillance and therapeutic planning. Adaptive learning frameworks may allow real-time updates as new data becomes available, improving clinical responsiveness. Embedding explainable ML tools into electronic health records and developing clinician-friendly interfaces will be key to adoption. Finally, ethical considerations such as transparency, patient communication, and equitable access must be addressed to ensure responsible implementation in oncological care.

## Ethics Statement

Ethical approval was obtained from the Helsinki University Hospital Institutional Review Board (HUS/491/2021) and the Finnish Medicines Agency (FIMEA/2021/005153).

## Funding Sources

This work was funded by Helsinki University Hospital research funds (TYH2020302) and 10.13039/501100010711Cancer Foundation Finland (WBS4708719), 10.13039/501100002301Estonian Research Council grant (PRG1076, MOB3JD1246, project nr 2021–2027.1.01.24-0750), and Horizon Europe (NESTOR, grant no. 101120075). The Finnish Cultural Foundation and K. Albin Johansson Foundation.

## CRediT authorship contribution statement

**Vijayachitra Modhukur:** Writing – review & editing, Writing – original draft, Supervision, Resources, Project administration, Funding acquisition, Formal analysis. **Mikko Loukovaara:** Writing – review & editing, Supervision, Resources, Project administration, Investigation, Data curation, Conceptualization. **Andres Salumets:** Writing – review & editing, Supervision, Resources, Project administration, Funding acquisition, Conceptualization. **Ralf Bützow:** Writing – review & editing, Supervision, Project administration, Funding acquisition, Conceptualization. **Annukka Pasanen:** Writing – review & editing, Formal analysis, Data curation, Conceptualization. **Masuma Khatun:** Writing – review & editing, Writing – original draft, Visualization, Formal analysis, Data curation, Conceptualization. **Sergio Vela Moreno:** Writing – original draft, Visualization, Validation, Software, Methodology, Investigation, Formal analysis, Conceptualization.

## Declaration of Competing Interest

None declared.

## Data Availability

The datasets used and/or analyzed during the current study are available from the corresponding author on reasonable request.

## Footnotes

Appendix A Supplementary data associated with this article can be found in the online version at doi:10.1016/j.csbj.2025.12.018.
