# Supplementary material for: Explainable machine learning for preoperative relapse prediction in molecularly stratified endometrial cancer: A single-center finnish cohort study
Source: Comput Struct Biotechnol J. 2025 Dec 22;31:143–56. doi: 10.1016/j.csbj.2025.12.018 (PMC12796588; doi:10.1016/j.csbj.2025.12.018)
Supplement: Supplementary file 1 — Supplementary material [file mmc1.docx]

**Supplementary References**

Aro, K., Loukovaara, M., Bützow, R., Pasanen, A., 2025. HER2 amplification and HER2 low expression in endometrial carcinoma: prevalence across molecular, histological and clinicopathological risk groups. BJC Reports 3. https://doi.org/10.1038/s44276-025-00125-6

Aro, K., Pasanen, A., Bützow, R., Loukovaara, M., 2024. The impact of estrogen receptor and L1 cell adhesion molecule expression on endometrial cancer outcome correlates with clinicopathological risk group and molecular subgroup. Gynecologic oncology 189, 9–15. https://doi.org/10.1016/j.ygyno.2024.06.016

Bartley, A.N., Washington, M.K., Colasacco, C., Ventura, C.B., Ismaila, N., Benson, A.B., Carrato, A., Gulley, M.L., Jain, D., Kakar, S., Mackay, H.J., Streutker, C., Tang, L., Troxell, M., Ajani, J.A., 2017. HER2 Testing and Clinical Decision Making in Gastroesophageal Adenocarcinoma: Guideline From the College of American Pathologists, American Society for Clinical Pathology, and the American Society of Clinical Oncology. Journal of Clinical Oncology 35, 446–464. https://doi.org/10.1200/jco.2016.69.4836

Guan, B., Mao, T.-L., Panuganti, P.K., Kuhn, E., Kurman, R.J., Maeda, D., Chen, E., Jeng, Y.-M., Wang, T.-L., Shih, I.-M., 2011. Mutation and loss of expression of ARID1A in uterine low-grade endometrioid carcinoma. The American journal of surgical pathology 35, 625–632. https://doi.org/10.1097/PAS.0b013e318212782a

Khatun, M., Pasanen, A., Kanerva, A., Koivisto-Korander, R., Tuomi, T., Bützow, R., Loukovaara, M., 2025. PPP2R1A mutation status as a predictive and prognostic factor in molecularly characterized endometrial carcinoma: a cohort study. International journal of gynecological cancer : official journal of the International Gynecological Cancer Society 35, 101934. https://doi.org/10.1016/j.ijgc.2025.101934

Lassus, H., Leminen, A., Lundin, J., Lehtovirta, P., Butzow, R., 2003. Distinct subtypes of serous ovarian carcinoma identified by p53 determination☆☆☆☆☆Supplementary data associated with this article can be found at doi: 10.1016/S0090-8258(03)00608-5. Gynecologic Oncology 91, 504–512. https://doi.org/10.1016/j.ygyno.2003.08.034

Meric-Bernstam, F., Makker, V., Oaknin, A., Oh, D.-Y., Banerjee, S., González-Martín, A., Jung, K.H., Ługowska, I., Manso, L., Manzano, A., Melichar, B., Siena, S., Stroyakovskiy, D., Fielding, A., Ma, Y., Puvvada, S., Shire, N., Lee, J.-Y., 2023. Efficacy and Safety of Trastuzumab Deruxtecan in Patients With HER2-Expressing Solid Tumors: Primary Results From the DESTINY-PanTumor02 Phase II Trial. Journal of Clinical Oncology: Official Journal of the American Society of Clinical Oncology 42, 101200JCO2302005. https://doi.org/10.1200/JCO.23.02005

Pasanen, A., Ahvenainen, T., Pellinen, T., Vahteristo, P., Loukovaara, M., Bützow, R., 2019. PD-L1 Expression in Endometrial Carcinoma Cells and Intratumoral Immune Cells. American Journal of Surgical Pathology 44, 174–181. https://doi.org/10.1097/pas.0000000000001395

Pasanen, A., Loukovaara, M., Ahvenainen, T., Vahteristo, P., Bützow, R., 2021. Differential impact of clinicopathological risk factors within the 2 largest ProMisE molecular subgroups of endometrial carcinoma. PLOS ONE 16, e0253472. https://doi.org/10.1371/journal.pone.0253472

Pasanen, A., Tuomi, T., Isola, J., Staff, S., Bützow, R., Loukovaara, M., 2016. L1 Cell Adhesion Molecule as a Predictor of Disease-Specific Survival and Patterns of Relapse in Endometrial Cancer. International Journal of Gynecologic Cancer 26, 1465–1471. https://doi.org/10.1097/igc.0000000000000801

Soovares, P., Pasanen, A., Similä-Maarala, J., Bützow, R., Lassus, H., 2022. Clinical factors and biomarker profiles associated with patient outcome in endometrioid ovarian carcinoma - Emphasis on tumor grade. Gynecologic oncology 164, 187–194. https://doi.org/10.1016/j.ygyno.2021.10.078

V. H. W. M. Jongen, Briët, J.M., R. De Jong, Klaske ten Hoor, Marike Boezen, van, Nijman, H.W., Hollema, H., 2009. Expression of estrogen receptor-alpha and -beta and progesterone receptor-A and -B in a large cohort of patients with endometrioid endometrial cancer. Gynecologic Oncology 112, 537–542. https://doi.org/10.1016/j.ygyno.2008.10.032
