# Supplementary material for: Explainable machine learning for preoperative relapse prediction in molecularly stratified endometrial cancer: A single-center finnish cohort study
Source: Comput Struct Biotechnol J. 2025 Dec 22;31:143–56. doi: 10.1016/j.csbj.2025.12.018 (PMC12796588; doi:10.1016/j.csbj.2025.12.018)
Supplement: Supplementary file 2 — Supplementary material [file mmc2.docx]

*Supplementary Figures*

**Explainable Machine Learning for Preoperative Relapse Prediction in Molecularly Stratified Endometrial Cancer: A Single-Center Finnish Cohort Study**

**Authors:** Sergio Vela Moreno^1,2¥^, Masuma Khatun^3,4¥^, Annukka Pasanen^4^, Ralf Bützow^3,4^, Andres Salumets^1,2,5^, Mikko Loukovaara^3,6*^, Vijayachitra Modhukur^1,2*β^

**Affiliation.** ^1^Department of Obstetrics and Gynecology, Institute of Clinical Medicine, University of Tartu, Tartu, Estonia, ^2^Celvia CC, Tartu, Estonia, ^3^Helsinki University Hospital and University of Helsinki, Department of Obstetrics and Gynecology, Helsinki, Finland, ^4^University of Helsinki, Faculty of Medicine, Helsinki University Hospital, and Research Program in Applied Tumor Genomics, Department of Pathology, Helsinki, Finland, ^5^Department of Clinical Science, Intervention and Technology, Karolinska Institute and Karolinska University Hospital, Stockholm, Sweden,
Finland, ^6^Helsinki University Hospital and University of Helsinki, Comprehensive Cancer Center, Helsinki, Finland

^¥^Shared first authorship; ^*^Shared last authorship; ^β^Corresponding author

^β^Corresponding author

Vijayachitra Modhukur, Department of Obstetrics and Gynecology, Institute of Clinical Medicine, University of Tartu, Tartu, Estonia, and Celvia CC, Tartu, Estonia

Email: vijayachitra.modhukur@ut.ee


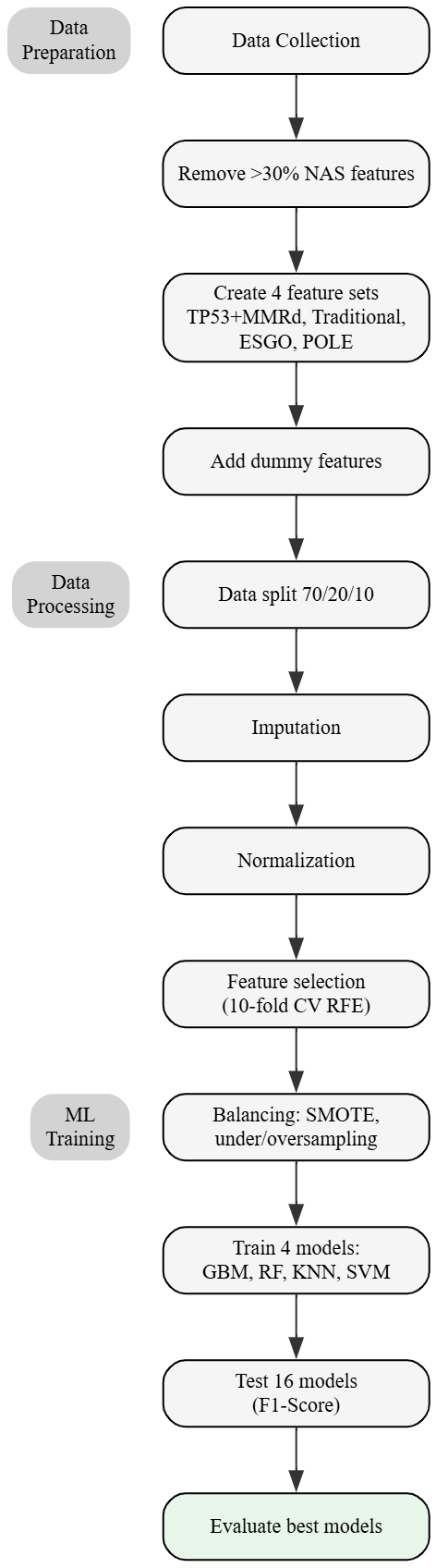


**Supplementary Figure S1**. **An Overview of the machine learning (ML) pipeline**. Flowchart depicts the study workflow, including data filtering (removing features with >30% missingness), cohort-specific feature set creation (TP53+MMRd, Traditional, ESGO, POLE), and preprocessing. The pipeline utilizes 10-fold cross-validation Recursive Feature Elimination (RFE) for stable feature selection, followed by model training (GBM, RF, KNN, SVM) and F1-score evaluation


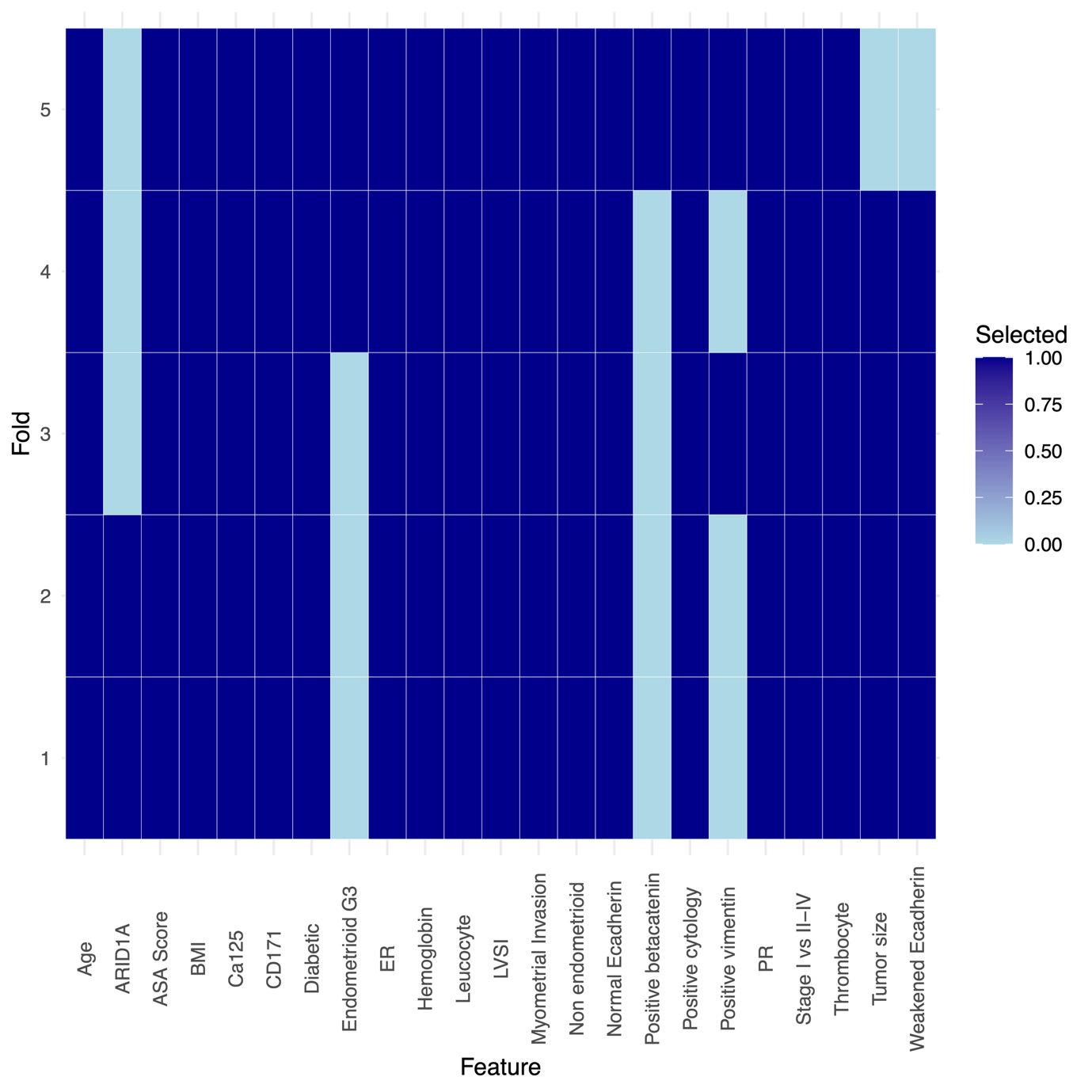


**Supplementary Figure S2**. **The recursive feature elimination stability in cross-validation folds**. Heatmap demonstrating the consistency of feature selection across cross-validation folds for the Traditional dataset. The y-axis represents the 5 folds, and the x-axis shows the top 20 most frequently selected features. Dark blue indicates feature selection within a specific fold, illustrating high stability for key clinical drivers such as FIGO stage, LVSI, and tumor size.


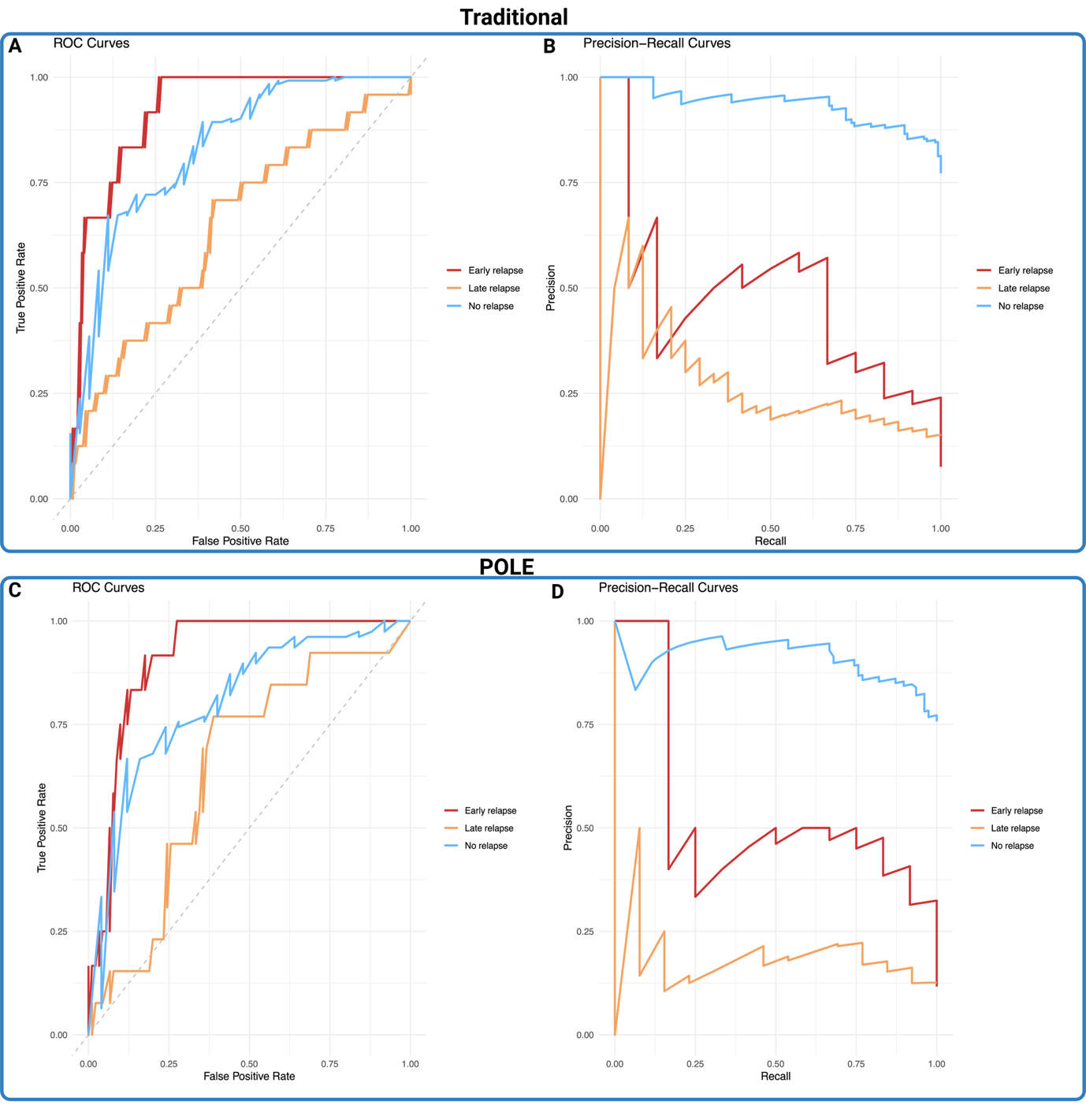


**Supplementary Figure S3**. **Recursive Feature Elimination stability per fold**. ROC-AUC and PR-AUC curves for the Traditional (A, B) and POLE (C, D) models. Curves represent multiclass prediction for Early, Late, and No relapse. Results show high predictive accuracy for "No relapse," while "Late relapse" presents the greatest challenge for classification across both model
